# Utility of Hounsfield unit in the diagnosis of tandem occlusion in acute ischemic stroke

**DOI:** 10.1007/s10072-020-04798-4

**Published:** 2020-10-14

**Authors:** Ruben Mühl-Benninghaus, Julia Dressler, Alena Haußmann, Andreas Simgen, Wolfgang Reith, Umut Yilmaz

**Affiliations:** Department of Neuroradiology, Saarland University Hospital, Kirrberger Straße, 66421 Homburg, Germany

**Keywords:** Stroke, Tandem occlusion, Large vessel occlusion, Computed angiography, Non-contrast head CT, Hounsfield

## Abstract

**Background:**

Tandem occlusions can complicate medical and endovascular stroke treatment. To identify these occlusions, computed tomography angiography (CTA) represents the best imaging modality. However, CTA is still not initially performed in some patients not admitted directly to stroke centers. Early identification of an additional occlusion of the proximal extracranial internal carotid artery may improve the best suitable treatment strategy. The purpose of this study was to find a valuable threshold of thrombus attenuation in a non-contrast head CT (NCCT) scan to facilitate a safe diagnosis of tandem occlusions.

**Materials and methods:**

Consecutive patients with acute middle cerebral artery (MCA) occlusions who underwent endovascular treatment were identified from our registry of neuroendovascular interventions. Thrombus attenuations of the affected MCA and contralateral vessel were measured by NCCT. To compare individual baseline blood attenuations, the difference between the thrombus attenuation and the contralateral MCA attenuation (referred to as ΔTM) was calculated.

**Results:**

Three hundred and twenty-five patients were included. There was a highly significant difference between mean thrombus attenuation with isolated MCA occlusion and additional extracranial internal carotid artery (ICA) occlusion (49.9 ± 8 vs. 56.2 ± 10 Hounsfield units (HU); *P* < 0.001). The area under the receiver operating characteristic curve of ΔTM was 0.72. The optimal threshold value was 13.5 HU, with a sensitivity of 67.5% and a specificity of 68.6%.

**Conclusion:**

Despite a significant difference in thrombus attenuation in MCA occlusions with an additional extracranial ICA occlusion compared with isolated MCA occlusions, a relevant threshold of thrombus attenuation was not found.

## Introduction

Endovascular treatment of acute intracranial artery occlusions has evolved rapidly. There are various treatment options, which include thrombolytic therapy, aspiration, or mechanical thrombectomy with a stent retriever. Information on the presence of thrombus expansion, e.g., tandem internal carotid artery (ICA)/middle cerebral artery (MCA) occlusion, is important for the outcome and material selection when performing mechanical recanalization [1–3]. In the anterior circulation, the hyperdense vessel sign on unenhanced (computed tomography) CT is an early and highly reliable indicator of the occlusive thrombus within the affected vessel [4]. However, the initial non-contrast head CT (NCCT) scan does not provide information on an additional proximal artery occlusion. A significantly higher thrombus attenuation was shown in patients with MCA occlusion and additional extracranial ICA occlusion when compared with MCA occlusion only [5].

Most hospitals performing CT in stroke patients usually implement computed tomography angiography (CTA) to confirm an occlusion and determine its location. However, based on our daily clinical practice, some stroke patients only undergo NCCT in the referring hospital. This is because of the lack of proper implementation of the CTA in the stroke imaging protocol or avoiding contrast administration due to kidney dysfunction in the patient. Information on thrombus attenuation may be a suitable tool to predict the expansion of the thrombus and, therefore, may aid in selecting the most suitable treatment modality, medically and/or endovascularly. Even if CTA is available, differentiation between true ICA occlusions and pseudo-occlusions can sometimes be difficult for the radiologist [6]. And even with duplex ultrasound, it can be hard to differentiate between subtotal and total occlusions of the ICA [7]. Consequently, measurement of thrombus attenuation may improve true ICA occlusion detection on unenhanced CT scans. Accordingly, reliable prediction of an additional extracranial ICA occlusion to improve stroke patient care is required. This retrospective study was conducted to assess the clinical benefits of attenuation measurement in patients with acute MCA occlusion as well as the value of a threshold for the diagnosis of an additional extracranial ICA occlusion.

## Materials and methods

The research in this study was conducted according to the principles of the Declaration of Helsinki. The local ethics committee approved the research protocol (12/17).

We performed a retrospective review of all consecutively endovascularly treated patients at our institution between 2009 and 2017. Inclusion criteria were an endovascular treated M1 segment occlusion and NCCT before digital subtraction angiography (DSA).

The imaging protocol for patients with suspected stroke in our department consists of a native CCT and CT angiography, followed by CT perfusion, which was performed on an Aquilion 32 Slice CT scanner (Toshiba Medical Systems, Tokyo, Japan) in helical mode (0.5-mm thickness, 120 kV). The native scan was reconstructed in the axial, sagittal, and coronal planes (0.5-mm thickness).

**Fig. 1:**
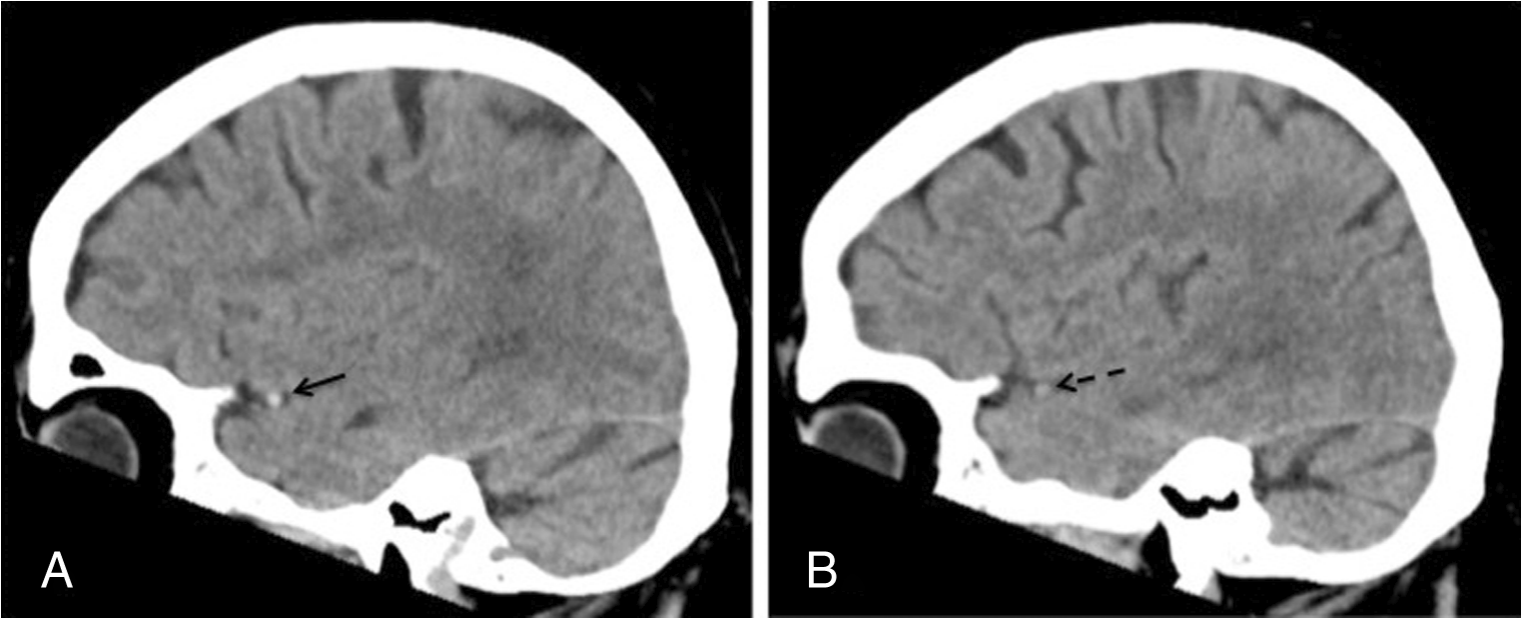
Sagittal reconstructions of a native CT scan of the same patient. **a** Thrombosis of MCA with a hyperdense sign (black arrow). **b** Freely perfused contralateral MCA (dashed arrow)

Thrombus attenuation and intraluminal attenuation of the contralateral MCA were measured in manually placed regions of interest perpendicular to the vessel on sagittal reconstructions of the CT scans (Fig. 1). To consider individual baseline blood attenuation in the statistical analysis, differences between the thrombus attenuation and the intraluminal attenuation of the contralateral MCA (referred to as ΔTM) were calculated. Additional extracranial ICA occlusions were noted on CTA and confirmed as true tandem occlusions on DSA.

Statistical analysis was performed using SPSS 25 (IBM, NY, USA). The *χ*^2^ test was applied to determine differences in the frequencies. Differences between the means were tested using Student’s *t* test. For both the attenuation and ΔTM, receiver operating characteristic (ROC) curves were obtained and analyzed to determine the optimal threshold by calculating the sensitivity and specificity. The significance level was defined as *P* < 0.05.

## Results

Data of 393 consecutive patients with acute occlusion of the M1 segment of the MCA who underwent endovascular treatment were analyzed retrospectively. Sixty-eight patients were excluded for the following reasons: initial CT scans were performed externally in the referring hospital (*n* = 55), retrospective analysis of the images was not possible (*n* = 4), an MRI was performed initially rather than a CT scan (*n* = 5), and the presence of a metal artifact close to the occluded MCA (*n* = 3). Spontaneous recanalization occurred in one patient.

From the remaining 325 patients, 162 were male and 163 female, with a mean age of 71 ± 13 years. Of these patients, data from 176 patients on the thrombus attenuation and angiographic results of mechanical thrombectomy with stent retrievers have been published previously [5]. The mean incidence rate of additional extracranial ICA occlusion was 25.5%. The highest rate was found in patients aged between 40 and 49 years (39%).

**Fig. 2:**
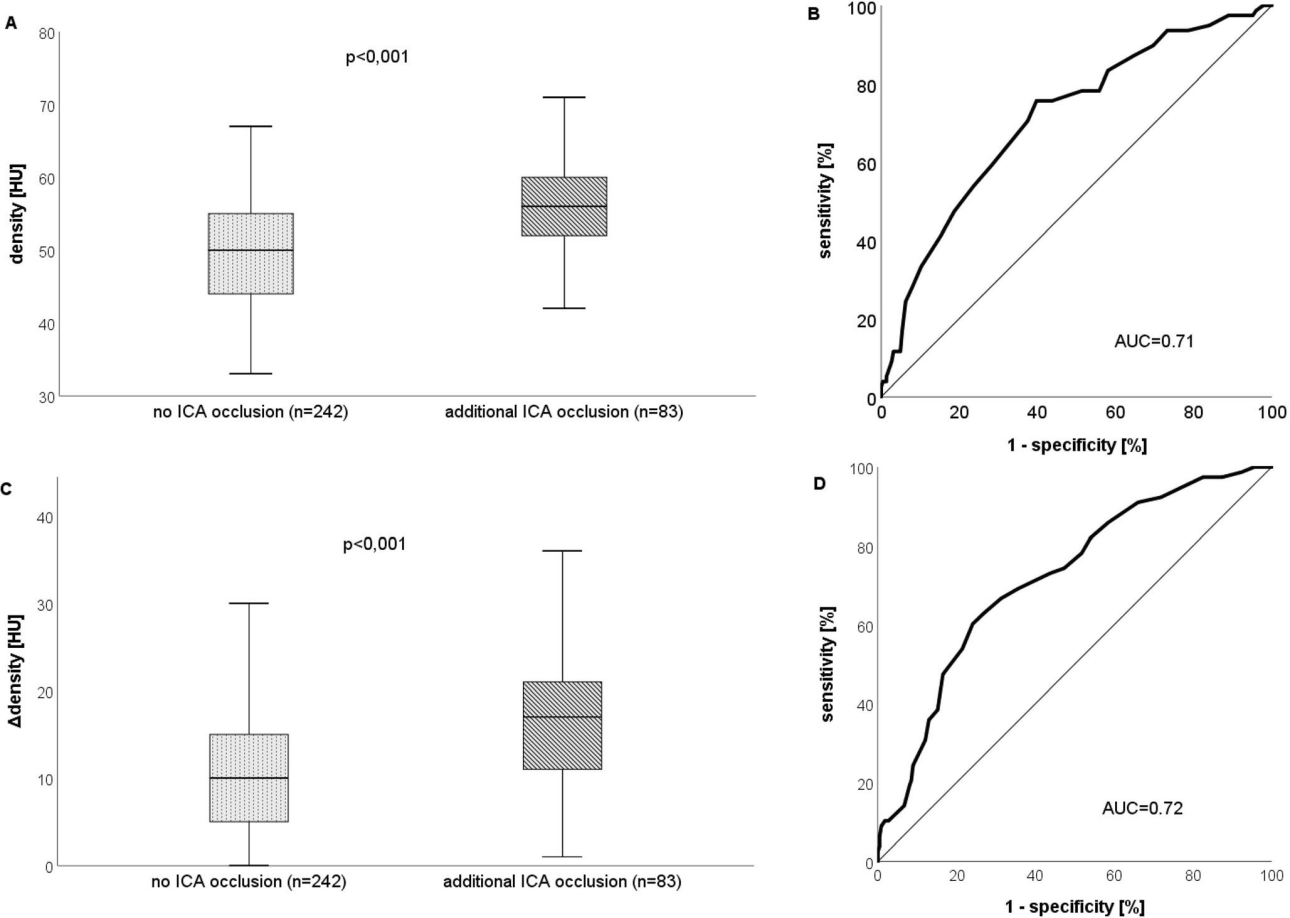
**a**, **c** Box plot demonstrating the mean and distribution of the attenuation values (density) among patients with an isolated MCA occlusion (no ICA) and additional occlusion of the extracranial ICA (ICA). Graph **a** shows statistics for the absolute attenuation values, whereas graph **c** demonstrates statistics of the relative values (ΔTM). **b**, **d** Receiver operating characteristic (ROC) curves and corresponding area under the curve (AUC) for the risk score of additional extracranial ICA occlusion. Graph **b** depicts statistics for the absolute attenuation values, whereas graph **c** shows statistics for relative values (ΔTM)

The mean thrombus attenuation was 6 Hounsfield units (HU) lower (95% CI 4–8 HU) in patients with isolated MCA occlusions (49.9 ± 8 HU) compared with patients with additional ICA occlusion (56.2 ± 10 HU) (Fig. 2a). These values are higher than the respective intraluminal densities of the contralateral MCA (39.2 ± 7 HU for isolated MCA occlusion, 38.2 HU for patients with tandem occlusion). This results in the mean difference ΔTM of 10.6 ± 8 HU for isolated occlusions and ΔTM of 17.8 ± 11 HU for patients with tandem occlusions. Thus, the ΔTM of patients with isolated MCA occlusions is significantly lower by 7 HU than for patients with tandem occlusions (95% CI 5–10 HU) (Fig. 2c). A total of 8 patients, belonging to both patient groups, had a higher attenuation of contralateral MCA than of that of the clots, resulting in a negative ΔTM. Calcified thrombi were observed in 8 (3.3%) patients with isolated MCA occlusions and in 2 (2.4%) patients with tandem occlusions.

**Fig. 3:**
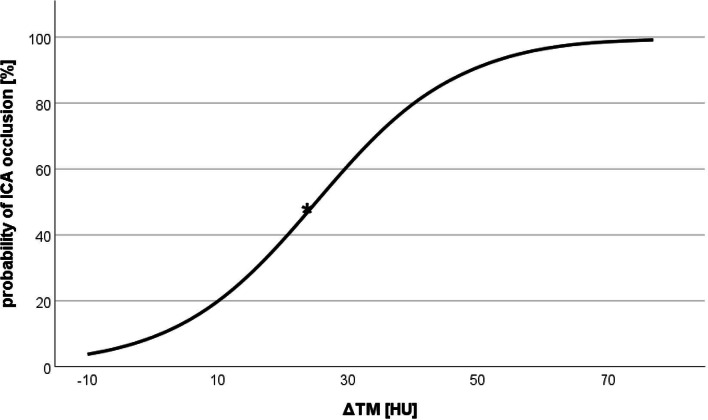
Logistic regression curve representing the estimated probability of an additional extracranial ICA occlusion, depending on ΔTM

ROC analysis (Fig. 2b, d) of thrombus attenuation in patients with an M1 occlusion and additional extracranial ICA occlusion showed an area under the curve (AUC) of 0.71 and an optimal threshold of 53.5 HU, with a sensitivity of 62.8% and a specificity of 68.7%. Furthermore, ROC analysis of the ΔTM of the thrombus attenuations and contralateral MCA attenuations showed an AUC of 0.72 and an optimal threshold of 13.5 HU. The resulting sensitivity was 67.5%, whereas the specificity was 68.6%. Logistic regression analyses (Fig. 3) of ΔTM > 25 HU showed a 50% probability of an additional extracranial ICA occlusion.

## Discussion

The presence of a hyperdense vessel sign in NCCT scans is a surrogate of an arterial occlusion by thrombus and represents an early ischemic change in acute stroke [8–10]. However, an NCCT scan alone cannot exclude an additional extracranial occlusion. In this study, we investigated whether measurement of thrombus attenuation in MCA occlusions is suitable for predicting an additional extracranial ICA occlusion. The early identification of tandem occlusion in acute stroke could be relevant to decide whether intravenous thrombolysis as bridging therapy may be required. To date, there is a continuous discussion about the increased risk of intracranial hemorrhage due to the bridging therapy and the additional administration of antiplatelet drugs (e.g., aspirin, clopidogrel, or tirofiban) in patients with tandem lesions when carotid stenting is needed [11, 12].

Our retrospective analysis confirmed that there is a significantly higher difference in thrombus attenuations and ΔTM in patients with additional occlusion of the extracranial ICA than in patients with isolated MCA occlusion [5]. The AUC for ΔTM was slightly higher than the curve for absolute HU values. Therefore, the mean differences in thrombus attenuations should be used for analysis.

Calcified thrombi were observed in a similar distribution in both analyzed patient groups with approximately 3%. These results are in line with previous reports [13, 14]. However, in eight cases, a negative ΔTM was found, which is attributable to a higher attenuation of contralateral MCA compared with those of the clots. A high lipid-rich composition might explain this preliminary observation but requires further exploration in larger sample–sized studies. Thrombus attenuation depends on the hematocrit and components such as cellular debris and lipids [15–17]. Furthermore, septic components of thrombi may influence the attenuation of thrombi [18].

The attenuation of red blood cell (RBC)–rich thrombi has been recently reported to be higher than that of fibrin-predominant thrombi [19, 20]. Cardioembolic thrombi have been hypothesized to contain a higher percentage of fibrin and fewer RBCs than non-cardioembolic thrombi [16, 21]. As arteriosclerotic events most likely cause tandem occlusions, our finding of higher thrombus attenuation in tandem occlusions is in agreement with these results. In contrast to these observations, Brinjikji et al. [20] found no significant difference in the proportion of RBC-rich thrombi between cardioembolic and large artery atherosclerosis etiologies. Further, Marder et al. [22] did not find differences in the histological composition of thrombus whether derived from cardiac or arterial sources in their series of 25 cases. However, larger prospective histopathological studies are required for confirmation of these findings.

The present study has several limitations. First, the study is restricted by its retrospective design. Second, due to the imbalance of the compared groups, the sample size was limited for a stroke study.

## Conclusion

Despite a significant difference in thrombus attenuation in MCA occlusions with an additional extracranial ICA occlusion compared with isolated MCA occlusions, a relevant threshold of thrombus attenuation was not found. Therefore, measurement of thrombus attenuation in MCA occlusions is not suitable for predicting an additional extracranial ICA occlusion. To identify such tandem occlusions, CTA remains the best clinical practice.

## Data Availability

Not applicable.

## Abbreviations

MCA: Middle cerebral artery
ICA: Internal carotid artery
NCCT: Non-contrast head CT scan
CTA: Computed tomography angiography
HU: Hounsfield unit
